# Association between serum calcium and prognosis in patients with acute pulmonary embolism and the optimization of pulmonary embolism severity index

**DOI:** 10.1186/s12931-020-01565-z

**Published:** 2020-11-11

**Authors:** Xin Wang, Yongbo Xiang, Ting Zhang, Yuqing Yang, Xuefeng Sun, Juhong Shi

**Affiliations:** 1 Department of Ultrasound, Peking Union Medical College Hospital, Beijing, China; 2 Department of Respiration, Peking Union Medical College Hospital, No. 1, Shuaifuyuan, Dongcheng District, Beijing, 100730 China; 3 Chinese Academy of Medical Sciences, Peking Union Medical College, Beijing, China; 4 Sogou Incorporated, Beijing, China

**Keywords:** Pulmonary thromboembolism, Serum calcium, Hypocalcemia, Prognosis, Pulmonary embolism severity index

## Abstract

**Introduction:**

Calcium is an important coagulation factor and hypocalcemia is related to progression and poor prognosis of many cardiopulmonary diseases. However, influence of hypocalcemia on pulmonary thromboembolism (PTE) prognosis has never been reported. This study aimed to explore its prognostic value and optimize the pulmonary embolism severity index (PESI), the widely used prognosis assessment model, based on the value.

**Methods:**

PTE patients’ variables in PESI and other related clinical characteristics including admission serum calcium were collected. Associations between these variables and PTE mortality were assessed by logistic regression and cox analysis. Variables significantly associated with 30-day PTE mortality were included to develop a new prognosis prediction rule and then its validity was compared with PESI and simplified PESI (sPESI).

**Results:**

496 PTE patients were included and 49.48% patients had hypocalcemia (serum calcium ≤ 2.13 mmol/L) in admission, showing higher 7-day (P = 0.021), 14-day (P = 0.002), 30-day (13.03% vs 4.98%, P = 0.002) mortalities than patients without hypocalcemia. Adjusting for variables in PESI, hypocalcemia was further revealed to be an independent predictor of 30-day mortality (P = 0.014).

The optimal prediction rule contained hypocalcemia and 5 variables in PESI and sPESI, showing higher predictive validity [sensitivity (Sen): 0.930, specificity (Spec): 0.390, area under curve (AUC): 0.800] than PESI (Sen: 0.814, Spec: 0.367, AUC: 0.716) and sPESI (Sen: 0.907, Spec: 0.216, AUC: 0.703).

**Conclusions:**

Hypocalcemia is an independent predictor of the mortality following acute PTE. Based on hypocalcemia, the optimal prediction rule showed higher validity than PESI and sPESI.

## Introduction

Acute pulmonary thromboembolism (PTE) is a life-threatening disease with high morbidity and mortality, which may cause an annual incidence rates ranging from 39 to 115 per 100,000 population and a 14-day mortality of 11.4% [1, 2]. Prognostic assessment of PTE patients, especially the evaluation of early death risk shows great significance for treatment.

The pulmonary embolism severity index (PESI), which includes 11 clinical parameters, is an extensively validated score in the prognostic assessment of PTE patients [3–5]. Owing to its complexity, a simplified version with 6 variables, known as simplified pulmonary embolism severity index (sPESI) was developed and validated [6, 7]. 2019 European guidelines recommended PESI or sPESI score to assess the prognosis and identify patients with low risk [1]. In addition, cardiac biomarkers and imaging testing are also used to further stratify patients with intermediate and high risk [8].

Calcium as the coagulation factor IV, participates in the coagulation process. Hypocalcemia, as a common electrolyte disorder in hospitalized patients, is related to the progression and poor prognosis of many cardiopulmonary diseases, such as heart failure, acute myocardial infarction, and acute exacerbations of chronic obstructive pulmonary disease [9–13]. Our previous exploratory research found that PTE patients with hypocalcemia had a higher death rate [14]. To date however, the influence of hypocalcemia on prognosis in patients with acute PTE has never been reported. This study aimed to explore the prognostic value of hypocalcemia on mortality following PTE and optimize the PESI.

## Methods

### Study design and participants

This is a single-center, retrospective, observational study. Consecutive inpatients enrolled met the inclusion criteria: aged 18 years or older, diagnosed with PTE in Peking Union Medical College Hospital (PUMCH) from January 1, 2012 to January 18, 2019. Patients were excluded if they were diagnosed and transferred from other health care facility due to lack of initial clinical data. Patients without important follow-up data were also excluded for the relevant mortality analysis. The diagnosis of PTE required to be confirmed with computed tomographic pulmonary angiography (CTPA), enhanced computed tomography of chest, scintigraphic ventilation-perfusion (V/Q) scan revealing high probability of PTE or to be diagnosed clinically by qualified specialist based on patients’ typical symptoms of PTE, finding deep venous thrombosis (DVT) in extremity by venous ultrasound/phlebography and positive D-dimer.

This study was approved by the Institutional Review Board of Peking Union Medical College Hospital (Ethical review number: B164), in accordance with the Declaration of Helsinki and also registered the clinical trial with identifiers of NCT04411888.

### Data collection

Eligible patients were searching according to the diagnosis code of PTE (ICD-Code: 126) in the hospital electronic medical record system. Patients identification and risk factors related to the prognosis of PTE were collected by three qualified doctors through the hospital electronic medical record system and reviewed by a specialist from pneumology department. The risk factors comprised variables in PESI, that is, age, gender, body temperature, pulse rate, respiratory rate, blood pressure, cancer, chronic heart failure, chronic pulmonary disease, altered mental status and arterial oxyhaemoglobin saturation, and other clinical characteristics, admission laboratory tests, imaging examinations, including dyspnea, chest pain, hemoptysis, syncope, surgery or trauma within 3 months, immobilization state, previous history of DVT, cardiopulmonary resuscitation (CPR) in hospital, glucocorticoid therapy history, hyperlipidemia or diabetes, hypertension, white blood cell, neutrophil proportion, hemoglobin, platelet, D-dimer, alanine aminotransferase (ALT), glutamyl transferase (GGT), albumin, creatinine, creatine kinase-Mb (CKMB), cardiac troponin I (cTnI), N-terminal B-type natriuretic peptide (NT-proBNP), serum calcium, serum potassium, serum sodium, serum chlorine, blood glucose, pH value, echocardiography et al. All the patients enrolled completed at least 1-month follow-up and confirmed the survival status as of January 18, 2019. The follow-up data was ascertained by interviewing patients, families or their physicians by means of telephone.

### Statistical analysis

Continuous and integer variables were presented as the mean value and SD for normally distributed variables, and the median and quartile for abnormal distributed variables. Categorical variables were expressed as their counts and proportions. Univariate logistic regression analysis was performed for variables in PESI and other admission laboratory indicators to assess the association between each factor and 7-day, 14-day, and 30-day PTE mortality respectively. Besides, part of continuous variables which were statistically significantly associated with the PTE mortality, were converted into categorical variables by selecting the maximum Youden index in receiver operating characteristic (ROC) curve as the cutoff value. Then associations between the newly generated categorical variables and PTE mortalities were analyzed.

Especially, patients were classified into two groups according to their serum calcium levels, that is, patients with or without hypocalcemia. Then differences between two groups were compared by two-sided independent Student's *t* test for normally distributed variables, Mann–Whitney U test for variables obeyed abnormal distribution, and chi-square test for categorical variables. Adjusting for variables in the PESI, cox analysis was adopted to determine the contribution of hypocalcemia to 30-day PTE mortality.

Then with 30-day all-cause mortality as the outcome, risk factors significantly associated with 30-day PTE mortality were included to develop a multivariate prognosis prediction rule. Notably considering the practicability, the categorical variables generated by continuous variables were included rather than continuous variables themselves. Each variable was assigned an integral point ranging from 0 to 5, and sum of all variables points was calculated to assess patient risk of death. Multiple prediction rules were generated by modifying variable points according to their significance. The rule with maximum area under curve (AUC) value was selected as the optimal prediction rule. By analyzing the ROC curve, the cutoff level of the optimal prediction rule was determined to identify low risk patients. To guarantee the simplicity and applicability, the building process tried to remove less effective variables with relatively lower points, without affecting the sensitivity and specificity of the prediction rule. More details of the building process could be found in the Additional file 3.

Sample size for multivariate prognosis prediction rule was estimated using tool from the website (https://mvansmeden.shinyapps.io/BeyondEPV/) [15] recommended by Riley et al. [16]. Validity of the optimal prediction rule, PESI, and sPESI was compared by computing their AUC, prevalence of low-risk patients, sensitivity, specificity, positive predictive value (PPV), negative predictive value (NPV), positive likelihood ratio (PLHR), and negative likelihood ratio (NLHR) [17]. The threshold of P-value was set to 0.05. Statistical analysis was conducted with IBM SPSS software (version 26.0, Inc, Chicago, IL, USA), Python 3.7.4, and RStudio 1.1.447.

## Results

### Baseline information

This study cohort comprised 496 patients with a median age of 61.5 (51, 70) years, and included 229 (46.17%) male patients. PTE was diagnosed in 440 patients (88.71%) by CTPA, V/Q scan in 35 (7.06%), enhanced computed tomography in 9 (1.81%) and clinical diagnosed in 12 (2.42%) patients. As of January 18, 2019, 361 patients survived and 135 died. The median survival time after diagnosis was 644.5 (218, 1693) days. The follow-up period of four patients were less than 30 days (1, 5, 11, and 20 days).

### Value of serum calcium in the prediction of PTE patients mortality

13 (2.62%) patients were excluded because of an undocumented serum calcium and a total of 483 patients were included for the following statistical analysis. Serum calcium levels ranged from 1.48 mmol/L to 2.60 mmol/L with a median value of 2.14 (2.04, 2.23) mmol/L. Univariate logistic regression analysis showed that serum calcium level was significantly associated with 7-day (β: 0.005, 95% CI 0.000–0063, P < 0.001), 14-day (β: 0.010, 95% CI 0.0001–0.087, P < 0.001), and 30-day mortalities (β: 0.035, 95% CI 0.0005–0.230, P = 0.001). Further by selecting 2.13 mmol/L as the cutoff value which had the maximum Youden index in ROC curve (Additional file 1: Fig. S1), patients with or without hypocalcemia were classified into two groups.

Overall, 239 (49.48%) patients with hypocalcemia (serum calcium level ≤ 2.13 mmol/L) showed significant higher 7-day (6.28% vs 2.06%, P = 0.021), 14-day (9.66% vs 2.89%, P = 0.002), 30-day (13.03% vs 4.98%, P = 0.002) mortalities. Adjusting for variables in PESI, cox regression analysis further revealed that hypocalcemia was an independent predictor of 30-day mortality (P = 0.014).

The clinical characteristics of the two groups were presented in Table 1. Distributions of age, gender, clinical symptoms on admission and most of important comorbidities were similar in two groups. Whereas, patients admitted with hypocalcemia had lower systolic and diastolic blood pressure (P = 0.031 and P = 0.036), faster pulse rate and respiratory rate (P = 0.012 and P < 0.001), higher body temperature (P < 0.001), higher shock index (P = 0.004) and more likely to have CPR in hospital (P = 0.014). As for laboratory parameters, patients with hypocalcemia had higher level of neutrophil proportion (P < 0.001), D-dimer (P < 0.001), CKMB (P = 0.005), cTnI (P = 0.046), NT-proBNP (P < 0.001), serum chlorine (P = 0.020), blood glucose (P < 0.001), GGT (P = 0.012) and lower level of hemoglobin (P < 0.001), platelet (P < 0.001), albumin (P < 0.001) and serum potassium (P = 0.031). Echocardiographic parameters were similar between two groups. Besides, patients with hypocalcemia had significantly higher PESI scores than the other group (P = 0.026), but there was no significant difference on their sPESI scores (P = 0.101).

**Table 1:** Clinical characteristics of 483 patients with acute PTE

Characteristics	All patients (n = 483)	Patients with hypocalcemia (serum calcium level ≤ 2.13 mmol/L, *n* = 239)	Patients without hypocalcemia (serum calcium level > 2.13 mmol/L, *n* = 244)	P value
Age(years)	62 (51, 70)	62 (51, 72)	61.5 (51, 69)	0.660
Male sex	224 (46.38%)	109 (45.61%)	115 (47.13%)	0.737
Symptoms on admission				
Dyspnea	313 (64.80%)	154 (64.44%)	159 (65.16%)	0.867
Chest pain	67 (13.87%)	28 (11.72%)	39 (15.98%)	0.175
Hemoptysis	45 (9.32%)	22 (9.21%)	23 (9.43%)	0.933
Syncope	53 (10.97%)	28 (11.72%)	25 (10.25%)	0.616
Comorbidities for PTE				
Chronic heart failure	138 (28.57%)	67 (28.03%)	71 (29.10%)	0.796
Altered mental status	81 (16.77%)	47 (19.67%)	34 (13.93%)	0.092
Sugery or trauma within 3 months	168 (36.05%)	89 (39.38%)	79 (32.92%)	0.146
Immobilization state	181 (38.92%)	89 (39.38%)	92 (38.49%)	0.845
Chronic pulmonary disease	140 (28.99%)	60 (25.10%)	80 (32.79%)	0.063
Cancer	169 (34.99%)	88 (36.82%)	81 (33.20%)	0.404
Previous history of DVT	295 (62.63%)	139 (60.43%)	156 (63.93%)	0.335
Underwent CPR in hospital	26 (5.39%)	19 (7.95%)	7 (2.88%)	**0.014**
Glucocorticoid therapy history	117 (25.05%)	65 (28.63%)	52 (21.67%)	0.082
Hyperlipidemia or diabetes	177 (37.98%)	88 (38.43%)	89 (37.55%)	0.846
Hypertension	198 (42.49%)	104 (46.02%)	94 (39.17%)	0.135
Physical examination findings				
Systolic blood pressure (mmHg)	122 (108, 135.25)	120 (104.75, 135)	123.5 (110, 136)	**0.031**
Diastolic blood pressure (mmHg)	74 (64, 82)	72 (61, 81)	75 (66, 83)	**0.036**
Pulse rate (b.p.m)	92 (80, 108)	94 (82, 110)	90 (78, 105)	**0.012**
Respiratory rate (/min)	20 (18, 22)	20 (18, 23)	20 (18, 20)	** < 0.001**
Temperature ( °C)	36.7 (36.4, 37.1)	36.8 (36.5, 37.3)	36.6 (36.4, 37.0)	** < 0.001**
Shock index	0.756 (0.625, 0.916)	0.790 (0.634, 0.977)	0.721 (0.613, 0.876)	**0.004**
Admission laboratory markers				
White blood cell (× 10^9^/L)	8.77 (6.29, 12.25)	8.98 (6.40, 12.60)	8.59 (5.99, 11.85)	0.260
Neutrophil proportion (%)	75.30 (66.13, 85.38)	79.9 (71.05, 87.25)	72.25 (63.25, 80.58)	** < 0.001**
Hemoglobin (g/L)	119.0 (99.5, 135.0)	109 (91, 130.25)	127 (113, 142)	** < 0.001**
Platelet (× 10^9^/L)	191 (138, 263)	179 (130, 241)	213 (150, 276)	** < 0.001**
D-dimer (mg/L)	4.98 (2.16, 11.45)	6.40 (3.33–16.40)	3.47(1.63–8.59)	** < 0.001**
ALT (U/L)	21 (14, 40)	20 (13, 47)	22 (14, 36)	0.858
GGT	49 (29.5, 99)	79.5 (33.5, 142.75)	42 (25, 66)	**0.012**
Albumin (g/L)	34 (30, 38)	30 (26, 34)	37 (34, 40)	** < 0.001**
Creatinine (umol/L)	69 (57, 85)	66 (54.75, 92.25)	70 (59, 82.5)	0.510
CKMB (ug/L)	0.90 (0.50, 1.90)	1.05 (0.50–2.20)	0.80 (0.50–1.50)	**0.005**
cTnI (ng/mL)	0.022 (0.009, 0.113)	0.030 (0.010, 0.181)	0.020 (0.006, 0.093)	**0.046**
NT-proBNP (pg/mL)	553 (148.5, 2086.25)	707 (207, 2909)	393 (104,1445)	** < 0.001**
Serum potassium (mmol/L)	3.9 (3.6, 4.2)	3.8 (3.5, 4.2)	3.9 (3.7, 4.2)	**0.031**
Serum sodium (mmol/L)	138 (136, 140)	138 (135, 140)	138 (136, 140)	0.213
Serum chlorine (mmol/L)	103 (100, 106)	104 (99, 107)	103 (100, 105)	**0.020**
Blood glucose	6.65 (5.50, 8.88)	7.25 (5.70, 9.80)	6.40 (5.30, 7.73)	** < 0.001**
PH	7.438 (7.405, 7.463)	7.440 (7.402, 7.471)	7.435 (7.407–7.457)	0.342
SaO_2_ (%)	94.00 (89.53, 96.88)	93.90 (89.00, 97.00)	94.30 (90.00, 96.50)	0.557
Echocardiography				
sPAP(mmHg)	57 (44, 71.5)	56 (41, 67)	57 (45.5, 72)	0.188
RVD	131 (29.91%)	59 (27.31%)	72 (32.43%)	0.282
PESI and sPESI				
PESI	99 (76, 127)	104 (79, 135)	98 (75, 119)	**0.026**
sPESI	1 (1, 2)	1 (1, 2)	1 (1, 2)	0.101

P values indicating statistically significant differences between patients with and without hypocalcemia are bolded

*Missing data*: 0.21% for Syncope, 3.52% for Surgery or trauma within 3 months, 3.73% for Immobilization state, 2.48% for Previous history of DVT, 0.21% for Underwent CPR in hospital, 3.31% for Glucocorticoid therapy history, 3.52% for Hyperlipidemia or Diabetes, 3.52% for Hypertension, 5.18% for Systolic blood pressure (mmHg), 5.18% for Diastolic blood pressure (mmHg), 3.31% for Pulse rate (b.p.m), 0.21% for White Blood Cell (× 109/L), 0.62% for Neutrophil proportion (%), 0.41% for Hemoglobin (g/L), 0.21% for Platelet (× 109/L), 3.11% for D-dimer (mg/L), 84.06% for GGT, 4.14% for Albumin (g/L), 1.86% for ALT (U/L), 1.66% for Creatinine (umol/L), 19.05% for CKMB, 14.08% for cTnI (ng/mL), 19.67% for NT-proBNP (pg/mL), 1.04% for Serum potassium (mmol/L), 0.62% for Blood glucose, 8.28% for PH, 8.90% for SaO_2_, 58.18% for sPAP(mmHg), and 42.86% for RVD

*PTE* pulmonary thromboembolism, *DVT* deep vein thrombosis, *CPR* cardiopulmonary resuscitation, *b.p.m* beats per minute, *ALT* alanine aminotransferase, *GGT* glutamyl transferase, *Cr* creatinine, *CKMB* creatine kinase-Mb, *cTnI* cardiac troponin I, *NT-proBNP* N-terminal pro-brain natriuretic peptide, *SaO*_*2*_ arterial oxygen saturation, *sPAP* systolic pulmonary artery pressure, *RVD* right ventricular dysfunction, *PESI* pulmonary thromboembolism severity index

### Association between other variables and PTE patients mortality

Other univariate associations between categorical variables with 7-day, 14-day, and 30-day mortality were shown in Table 2. For variables of the PESI or sPESI, 5 variables, including "systolic blood pressure (BP) < 100 mmHg", "pulse rate ≥ 110 beats per minute (b.p.m)", "respiratory rate > 30 breaths per min", "altered mental status", and "chronic heart failure" were all significantly associated with 7-day, 14-day, and 30-day mortality. "Age" was associated with 7-day and 14-day mortality, while "age > 80 years" was associated with 14-day and 30-day mortality. "History of heart failure or chronic pulmonary disease" was associated with 7-day and 30-day mortality. Besides, there was association between "cancer" and 30-day mortality. "SaO_2_ < 90%" was only associated with 7-day mortality. Other variables including "male sex", "temperature < 36 °C", and "chronic pulmonary disease" had no significant associations with mortalities. Among other laboratory examination variables, serum calcium, NT-proBNP, D-dimer and blood glucose had significant correlations with 7-day, 14-day, and 30-day mortalities. After converting these four variables into the categorical variables (Additional file 1: Fig. S1), hypocalcemia, high NT-proBNP, high D-dimer were still associated with 7-day, 14-day, and 30-day mortalities, and high blood glucose was associated with 7-day mortality.

**Table 2:** Univariate associations between variables from PESI, sPESI, and other laboratory tests and 7-day, 14-day, and 30-day PTE mortalities

Variable Name	All patients (n = 496)	7-day Mortality {β (95% CI) [P-value]}	14-day Mortality {β (95% CI) [P-value]}	30-day Mortality {β (95% CI) [P-value]}
N	–	4.45% (22/494)	6.49% (32/493)	9.15% (45/492)
Variables from PESI or sPESI				
Age(years)	61.5 (51, 70)	1.043 (1.008, 1.079) [0.014]	1.044 (1.015, 1.074) [0.003]	1.018 (0.997, 1.039) [0.100]
Age > 80 years	5.04% (25/496)	3.230 (0.888, 11.740) [0.075]	6.891 (2.635, 18.025) [< 0.001]	4.390 (1.725, 11.171) [0.002]
Male sex	46.17% (229/496)	1.410 (0.598, 3.327) [0.433]	1.523 (0.740, 3.136) [0.253]	1.489 (0.804, 2.759) [0.206]
Systolic BP < 100 mmHg	15.52% (77/496)	6.152 (2.564, 14.761) [< 0.001]	4.244 (1.998, 9.014) [< 0.001]	3.916 (2.022, 7.585) [< 0.001]
Pulse rate ≥ 110 b.p.m	22.18% (110/496)	4.580 (1.922, 10.911) [0.001]	3.399 (1.638, 7.054) [0.001]	3.570 (1.900, 6.705) [< 0.001]
Temperature < 36 °C	3.63% (18/496)	–	0.897 (0.115, 6.989) [0.917]	0.612 (0.079, 4.727) [0.638]
Respiratory rate > 30 breaths per min	6.05% (30/496)	3.812 (1.203, 12.079) [0.023]	4.202 (1.580, 11.176) [0.004]	2.712 (1.046, 7.031) [0.040]
SaO_2_ < 90%	23.19% (115/496)	3.583 (1.510, 8.498) [0.004]	2.112 (0.999, 4.465) [0.050]	1.587 (0.813, 3.101) [0.176]
Altered mental status	17.34% (86/496)	12.306 (4.844, 31.264) [< 0.001]	6.550 (3.124, 13.735) [< 0.001]	5.236 (2.754, 9.957) [< 0.001]
Chronic heart failure	28.43% (141/496)	3.191 (1.346, 7.565) [0.008]	2.346 (1.137, 4.838) [0.021]	2.155 (1.155, 4.022) [0.016]
Chronic pulmonary disease	28.63% (142/496)	1.765 (0.737, 4.226) [0.202]	1.761 (0.845, 3.669) [0.131]	1.737 (0.924, 3.265) [0.087]
Cancer	34.88% (173/496)	1.300 (0.544, 3.106) [0.555]	1.709 (0.831, 3.512) [0.145]	1.913 (1.033, 3.544) [0.039]
History of chronic heart failure or chronic pulmonary disease	42.54% (211/496)	3.017 (1.208, 7.539) [0.018]	2.048 (0.987, 4.246) [0.054]	1.938 (1.042, 3.605) [0.037]
Continuous variables from laboratory tests				
Serum calcium	2.14 (2.04, 2.23)	0.005 (0.000, 0063) [< 0.001]	0.010 (0.001, 0.087) [< 0.001]	0.035 (0.005, 0.230) [0.001]
Calcium (albumin adjustment)	2.29 (2.22, 2.38)	0.397 (0.009, 18.354) [0.637]	0.767 (0.038, 15.390) [0.862]	0.923 (0.078, 10.859) [0.949]
cTnI	0.0235 (0.0090, 0.1190)	1.017 (0.962, 1.076) [0.546]	1.020 (0.974, 1.068) [0.402]	1.012 (0.965, 1.060) [0.629]
NT-proBNP	561 (150, 2114.75)	1.000 (1.000, 1.000) [0.021]	1.000 (1.000, 1.000) [0.002]	1.000 (1.000, 1.000) [0.009]
D-dimer	4.96 (2.16, 11.43)	1.019 (1.006, 1.033) [0.005]	1.018 (1.006, 1.031) [0.004]	1.013 (1.001 1.025) [0.034]
GGT	49 (29.5, 99)	0.965 (0.909, 1.026) [0.253]	0.991 (0.969, 1,013) [0.414]	0.999 (0.991, 1.008) [0.871]
Cr(umol/L)	69 (57, 85)	1.001 (0.997, 1.004) [0.703]	1.000 (0.996, 1.004) [0.967]	1.001 (0.999, 1.003) [0.473]
Blood glucose (mmol/L)	6.6 (5.5, 8.875)	1.115 (1.034, 1.203) [0.005]	1.105 (1.032, 1.182) [0.004]	1.081 (1.015, 1.152) [0.015]
Serum potassium (mmol/L)	3.9 (3.6, 4.2)	2.008 (0.907, 4.443) [0.085]	1.484 (0.752, 2.931) [0.255]	1.455 (0.817, 2.594) [0.203]
Serum sodium(mmol/L)	138 (136, 140)	0.979 (0.882, 1.087) [0.688]	0.959 (0.880, 1.044) [0.332]	0.937 (0.872, 1.006) [0.074]
Serum chlorine(mmol/L)	103 (100,106)	0.992 (0.905, 1.089) [0.873]	0.991 (0.918, 1.070) [0.815]	0.961 (0.902, 1.023) [0.215]
Categorical variables by thresholding significantly associated continuous variables from laboratory tests				
Hypocalcemia	49.48% (239/483)	3.174 (1.135, 8.877) [0.028]	3.591 (1.511, 8.538) [0.004]	2.858 (1.430, 5.711) [0.003]
High NT-proBNP	38.64% (153/396)	4.109 (1.418, 11.909) [0.009]	2.750 (1.213, 6.231) [0.015]	2.570 (1.288, 5.128) [0.007]
High D-dimer	63.45% (302/479)	5.862 (1.349, 25.474) [0.018]	5.880 (1.761, 19.638) [0.004]	3.237 (1.424 7.524) [0.005]
High blood glucose (mmol/L)	43.18% (209/484)	2.566 (1.005, 6.550) [0.049]	2.079 (0.978, 4.420) [0.057]	1.753 (0.933, 3.296) [0.081]

*BP* blood pressure, *b.p.m* beats per minute, *SaO*_*2*_ arterial oxygen saturation, *cTnI* cardiac troponin I, *NT-proBNP* N-terminal pro-brain natriuretic peptide, *GGT* glutamyl transferase, *Cr* creatinine

### The derivation and validation of the prediction rule

12 categorical variables significantly associated with 30-day mortality were used to build the prediction rules (see Additional file 2: Fig. S2 and Additional file 3 for details). Minimally required total sample size was 450 and minimally required events per variable (EPV) was 3 for 12 variables by setting events fraction, or 30-day mortality, to 0.08, which were met in this study. Finally, 6 variables were included into the optimal prediction rule shown in Table 3. In the optimal prediction rule, sum of variables points no less than 4 points was identified as the high risk.

**Table 3:** Comparisons of the optimal prediction rule, PESI and sPESI

Variable	PESI	sPESI	Optimal Prediction rRule
Age	Age in years	1 point (if age > 80 years)	4 points (if age > 80 years)
Male sex	10 points		
Cancer	30 points	1 point	4 points
Chronic heart failure	10 points	1 point	2 points
Chronic pulmonary disease	10 points		
Pulse rate ≥ 110b.p.m	20 points	1 point	4 points
Systolic BP < 100 mmHg	30 points	1 point	
Respiratory rate > 30 breaths per min	20 points		
Temperature < 36 °C	20 points		
Altered mental status	60 points		5 points
Arterial oxyhaemoglobin saturation < 90%	20 points	1 point	
Serum calcium ≤ 2.13 mmol/L			3 points

In the optimal prediction rule, sum of points of six variables ≥ 4 was identified as the high death risk

**Fig. 1:**
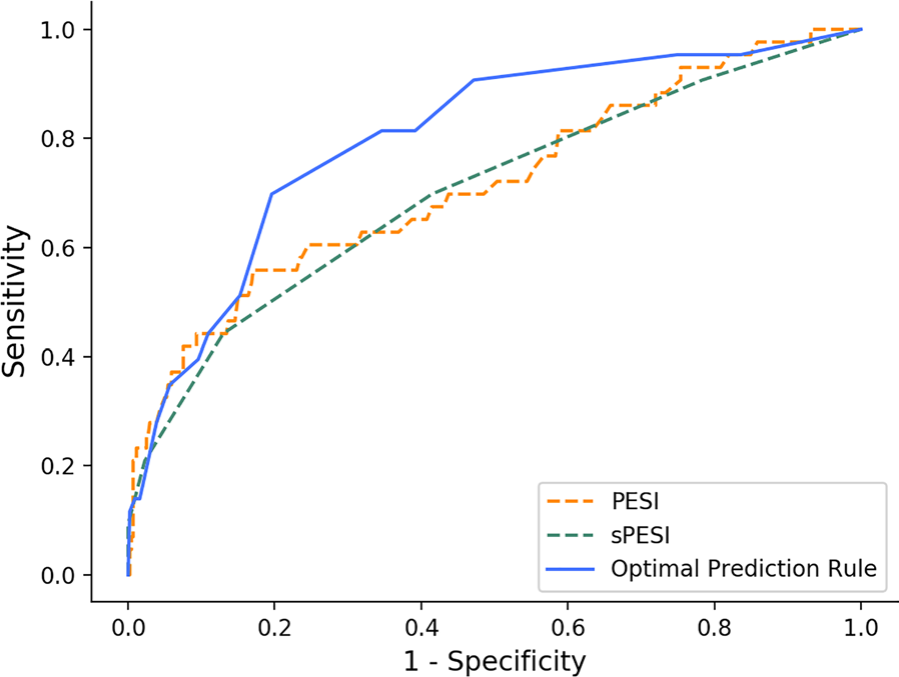
Comparisons of ROC curves of PESI, sPESI, and the optimal prediction rule

In the whole dataset, ROC curves of PESI, sPESI and the optimal prediction rule were shown in Fig. 1. ROC curve of the optimal prediction rule was close to the PESI and sPESI on the beginning, and then climbed obviously higher than them. Validity of three rules was listed at Table 4. Prevalence of 30-day mortality was 0.089 (95% CI 0.065, 0.118). Generally, mean values of AUC, sensitivity (Sen), specificity (Spec), of the optimal prediction rule (Sen: 0.930, Spec: 0.390, AUC: 0.800) were better than both the PESI (Sen: 0.814, Spec: 0.367, AUC: 0.716) and sPESI (Sen: 0.907, Spec: 0.216, AUC: 0.703), although their 95% CIs might overlap. The optimal rule also had advantages of PPV, NPV, PLHR, and NLHR over PESI and sPESI. Especially, specificity and positive likelihood ratio of the optimal prediction rule were significantly higher than the sPESI.

**Table 4:** Comparison of predictive validity of the optimal prediction rule to the PESI and sPESI

Characteristic	Optimal (95% CI)	PESI (95% CI)	sPESI (95% CI)
AUC	0.800 (0.729, 0.871)	0.716 (0.626, 0.805)	0.703 (0.613, 0.793)
Prevalence of low-risk	0.639 (0.594, 0.682)	0.649 (0.605, 0.692)	0.795 (0.756, 0.830)
Sensitivity	0.930 (0.809, 0.985)	0.814 (0.666, 0.916)	0.907 (0.779, 0.974)
Specificity	0.390 (0.344, 0.437)	0.367 (0.322, 0.414)	0.216 (0.179, 0.258)
Positive predictive value	0.130 (0.094, 0.173)	0.112 (0.079, 0.152)	0.102 (0.073, 0.137)
Negative predictive value	0.983 (0.950, 0. 996)	0.953 (0.909, 0.979)	0.960 (0.900, 0.989)
Positive likelihood ratio	1.524 (1.364, 1.702)	1.285 (1.096, 1.508)	1.157 (1.039, 1.289)
Negative likelihood ratio	0.179 (0.060, 0.537)	0.507 (0.268, 0.959)	0.430 (0.166, 1.112)

*CI* confidence interval

## Discussion

Our results demonstrated that hypocalcemia (serum calcium ≤ 2.13 mmol/L) was present in a substantial proportion of acute PTE patients (49.48%) at the time of admission and PTE mortality were higher in patients with lower serum calcium. To our knowledge, it is the first to explore the prognostic importance of hypocalcemia in patients with acute PTE. Based on its prognostic predictive value, we proposed the optimal prediction rule, showing higher sensitivities and specificities than PESI and sPESI.

Calcium is an important cofactor of the coagulation cascade and might therefore participates in the pathophysiology of severe thromboembolic and hemorrhagic events. Hypocalcemia has been reported in patients with ST-segment elevation myocardial infarction, intracerebral hemorrhage, and infants with hypoxic-ischemic encephalopathy [18–22]. Nevertheless, there is no literature reports about the correlation between hypocalcemia and PTE. Except for Usta et al. reported a 56-year old female had PTE after total hip replacement, presumably triggered by hypocalcemia [23]. There are several possible explanations for the association between hypocalcemia and PTE. One possible mechanism is related to platelet activation, which happened in the process of thrombus formation in PTE patients [24, 25]. Since the entry of extracellular Ca^2+^ through plasma membrane, leading to the reduction of serum calcium [26], is a major source of the increased intracellular Ca^2+^ concentration during platelet activation, hypocalcemia may indicate the activation of platelet in PTE patients. Another possible mechanism is related to hypoxic pulmonary vasoconstriction (HPV), which may lead Ca^2+^ enter from extracellular environment by activating channels like voltage-dependent Ca^2+^ channels (VDCCs), store-operated channels (SOC), receptor-operated channels (ROC), and acid-sensing ion channel 1a (ASIC1a) [27–29]. Ca^2+^ influx can further activate vasoconstriction and lead to pulmonary hypertension [29]. Therefore, hypocalcemia might occur in the process of HPV.

Our optimal prediction rule showed certain consistency with PESI and sPESI. Four variables associated with PTE prognosis, age > 80 years, pulse rate, heart failure, and cancer, were all included in these three rules, which indicated the prediction rule's reasonability. Besides, our prediction rule differed from PESI and sPESI in some aspects. Comparing with sPESI, the prediction rule comprised "altered mental status" as an important variable and also assigned different points to different variables in order to reflect the diagnostic values of different variables better. Variable points in our prediction rule were relatively consistent with PESI, including assigning higher points to "altered mental status", "cancer", "pulse rate ≥ 110b.p.m" and "age > 80 years". Comparing with PESI, our prediction rule was more simplified and meanwhile kept high predictive ability. Further, as a common clinical indicator and the newly added variable, hypocalcemia showed high prognostic value and helped to improve the rule's predictive validity. Meanwhile, it is also economic and easy to get even in the emergency room, showing high health economics value.

Several clinical and research implications could be practiced based on our findings. More intensive surveillance of serum calcium for PTE patients should be adopted in admission. Further studies are expected to explore the mechanism of hypocalcemia and whether PTE patients with low serum calcium levels could benefit from calcium supplement. Additionally, the optimal prediction rule proposed in this study may be helpful to assess the prognosis of patients with acute PTE, but still needs to be validated in larger datasets.

Our work has several limitations. First, 13 (2.62%) patients were excluded because serum calcium was not measured at admission. Second, our data can only analysis the correlation between hypocalcemia and the prognosis of PTE, not the causal relationship. Thus, we cannot determine the specific role that serum calcium plays in the pathophysiology of PTE. Third, multi-center studies with larger sample size are also needed to verify the rule's validity.

## Conclusion

Hypocalcemia is widespread in patients with acute PTE. Our study presented for the first time that admission hypocalcemia was an independent predictor of the 30-day mortality following acute PTE. Based on PESI and hypocalcemia, our optimal prediction rule showed better prognostic predictive performance than PESI and sPESI.

## Supplementary information

**Additional file 1: Figure S1.** ROC curves for 30-day mortality for the serum calcium, NT-proBNP, D-dimer, and blood glucose.

**Additional file 2: Figure S2.** Comparison of ROC curves of PESI, sPESI and the prediction rule using 12 variables.

**Additional file 3.** Detailed process of derivation and validation of the prediction rule.

## Data Availability

All the data will be available to other researchers on reasonable requests to the corresponding author after publication.

## Abbreviations

PTE: Pulmonary thromboembolism
PESI: Pulmonary embolism severity index
sPESI: Simplified pulmonary embolism severity index
Sen: Sensitivity
Spec: Specificity
AUC: Area under curve
PUMCH: Peking Union Medical College Hospital
CTPA: Computed tomographic pulmonary angiography
V/Q: Ventilation–perfusion
DVT: Deep venous thrombosis
CPR: Cardiopulmonary resuscitation
ALT: Alanine aminotransferase
GGT: Glutamyl transferase
CKMB: Creatine kinase-Mb
cTnI: Cardiac troponin I
NT-proBNP: N-terminal B-type natriuretic peptide
ROC: Receiver operating characteristic
PPV: Positive predictive value
NPV: Negative predictive value
PLHR: Positive likelihood ratio
NLHR: Negative likelihood ratio
CI: Confidence interval
