# Supplementary figures and images for: Association between serum calcium and prognosis in patients with acute pulmonary embolism and the optimization of pulmonary embolism severity index
Source: Respir Res. 2020 Nov 11;21:298. doi: 10.1186/s12931-020-01565-z (PMC7659049; doi:10.1186/s12931-020-01565-z)

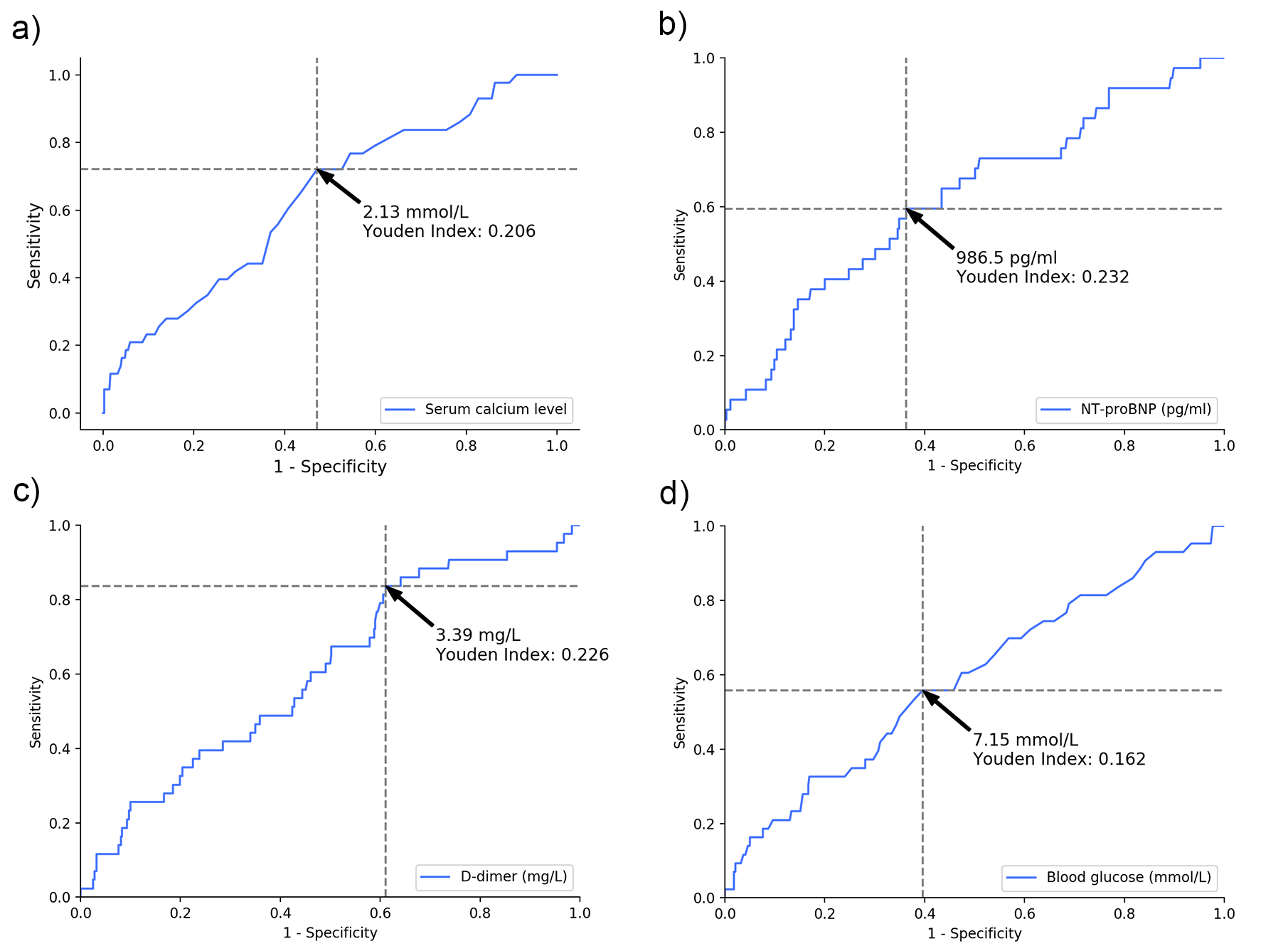

Supplement: Supplementary file 1 — Additional file 1: Figure S1. ROC curves for 30-day mortality for the serum calcium, NT-proBNP, D-dimer, and blood glucose. [file 12931_2020_1565_MOESM1_ESM.tif]

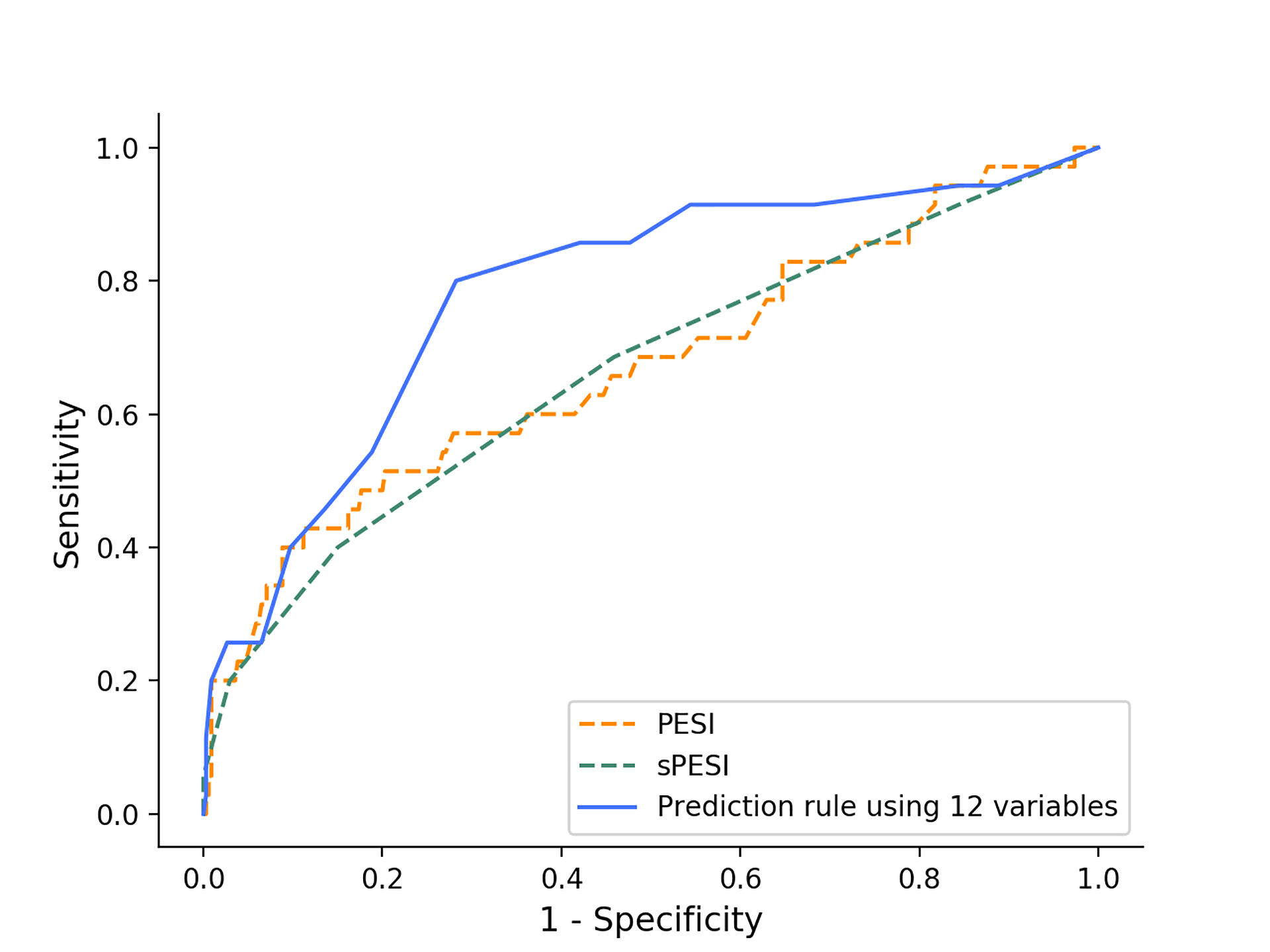

Supplement: Supplementary file 2 — Additional file 2: Figure S2. Comparison of ROC curves of PESI, sPESI and the prediction rule using 12 variables. [file 12931_2020_1565_MOESM2_ESM.tif]
