# Supplementary material for: Association between serum calcium and prognosis in patients with acute pulmonary embolism and the optimization of pulmonary embolism severity index
Source: Respir Res. 2020 Nov 11;21:298. doi: 10.1186/s12931-020-01565-z (PMC7659049; doi:10.1186/s12931-020-01565-z)
Supplement: Supplementary file 3 — Additional file 3. Detailed process of derivation and validation of the prediction rule. [file 12931_2020_1565_MOESM3_ESM.docx]

**Detailed process of derivation and validation of the prediction rule**

**Converting continuous variables into the categorical variables**

By univariate analysis, except for the serum calcium, three continuous variables including NT-proBNP, D-dimer, and blood glucose, were significantly associated with the 30-day mortality. For the convenience in clinicals, we converted them into the categorical variables by analyzing their ROC curves and selecting the value with the maximum Youden index as the cutoff level. ROC curves of three variables were shown in **Figure S1** with corresponding cutoff levels annotated.

**Building the optimal prediction rule using categorical variables**

12 categorical variables significantly associated with 30-day mortality were first included to build the multivariate risk prediction rule. Each variable could be assigned one of 6 integer points (0, 1, 2, 3, 4, and 5) and the sum of all variables’ points was calculated to assess the 30-day death risk. Different combinations of points of variables were considered and rule with maximum AUC value by the ROC analysis was selected, which was achieved by using the ‘Hyperopt’ package in Python 3.7.4.

The prediction rule using 12 variables with the maximum AUC was shown in **Table S1** and corresponding ROC curve was plotted in **Figure S2**, which also showed the ROC curves of PESI and sPESI. For the prediction rule using 12 variables, 4 points was selected as the cutoff level to identify high-risk patients and its sensitivity and specificity were listed in **Table S2**.

As we can see, its sensitivity was higher than PESI and similar with sPESI. Its specificity was superior to both PESI and sPESI. Considering the fact that "NT-probBNP", "D-dimer", and "blood glucose" had relatively low points, for the simplicity and applicability of prediction rule, we tried to remove them to build a new prediction rule.

**Table S1. The prediction rules using 12 variables and 9 points with the maximum AUC value**

| **Variable Name** | **Points of the prediction rule with 12 variables** | **Points of the prediction rule with 9 variables** |
| --- | --- | --- |
| Age>80 years | 4 | 4 |
| Systolic BP <100 mmHg | 0 | 0 |
| Pulse rate ≥110 b.p.m. | 2 | 4 |
| Respiratory rate >30 breaths per min | 1 | 0 |
| SaO_2_ <90% | 0 | 0 |
| Altered mental status | 4 | 5 |
| Chronic heart failure | 3 | 2 |
| Cancer | 3 | 4 |
| Hypocalcemia | 4 | 3 |
| NT-proBNP (pg/mL) | 1 | -- |
| D-dimer (mg/L) | 0 | -- |
| Blood glucose (mmol/L) | 0 | -- |

**Table S2. Predictive validity of sPESI, PESI and the prediction rule using 12 variables**

| **Rule Name** | **Sensitivity** | **Specificity** | **AUC** |
| --- | --- | --- | --- |
| sPESI | 0.914 | 0.156 | 0.665 |
| PESI | 0.829 | 0.297 | 0.672 |
| The prediction rule using 12 variables | 0.914 | 0.318 | 0.781 |

By setting points of "NT-probBNP", "D-dimer", and "blood glucose" into zero, we rebuilt the prediction rule. The prediction rule using 9 variables were listed at **Table S1**, and comparisons of the validity of three models were shown in **Figure 2** and **Table S3**. After removing these three variables, the new prediction rule using 9 variables achieved higher sensitivity, specificity, and AUC score than the PESI and sPESI. In addition, we also compared the prediction rule using 9 variables to the previous prediction rule using 12 variables, which was shown in **Table S4**. Higher sensitivity and specificity were achieved by the prediction rule with 9 variables.

Notably, in this prediction rule using 9 variables, points of "systolic BP <100 mmHg", "respiratory rate >30 breaths per min", and "SaO_2_ <90%" were all zeros, so there were only 6 non-zero variables, that is, the final optimal prediction rule.

**Table S3. Predictive validity of sPESI, PESI and the prediction rule using 9 variables**

| **Rule Name** | **Sensitivity** | **Specificity** | **AUC** |
| --- | --- | --- | --- |
| sPESI | 0.907 | 0.216 | 0.703 |
| PESI | 0.814 | 0.367 | 0.716 |
| The prediction rule using 9 variables | 0.930 | 0.390 | 0.800 |

**Table S4. Comparison of validity of prediction rules using 12 and 9 variables**

| **Rule Name** | **Sensitivity** | **Specificity** | **AUC** |
| --- | --- | --- | --- |
| sPESI | 0.914 | 0.156 | 0.665 |
| PESI | 0.829 | 0.297 | 0.672 |
| The prediction rule using 12 variables | 0.914 | 0.318 | 0.781 |
| The prediction rule using 9 variables | 0.943 | 0.332 | 0.771 |
